# Improvement in near-infrared absorbance attenuation by using nanometer black silicon composited with gold nanoparticles

**DOI:** 10.1186/s11671-023-03847-z

**Published:** 2023-06-03

**Authors:** Guanyu Mi, Jian Lv, Longcheng Que, Cheng Tan, Jian Huang, Zhongyuan Liu, Lintong Zhao

**Affiliations:** 1 State Key Laboratory of Electronic Thin Film and Integrated Devices, University of Electronic Science and Technology of China, Chengdu, 610054 China; 2 China Electronics Technology Group Corporation Chongqing Acoustic Optic Electronic CO., LT, Chongqing, 401332 China

**Keywords:** Nanometer black silicon, Gold nanoparticles, Near-infrared absorption, Broad spectrum

## Abstract

In order to solve the problem of near-infrared (NIR) absorbance attenuation of silicon, a method of preparing gold nanoparticles (AuNPs) on the micro–nano-structured black silicon (B-Si) is proposed. In this study, the local surface plasmon resonance (LSPR) of AuNPs excited by a light field is used to achieve B-Si materials with broad spectrum and high absorption. The results show that nanometer B-Si composited with 25-nm AuNPs has an average absorption of 98.6% in the spectral range of 400–1100 nm and 97.8% in the spectral range of 1100–2500 nm. Compared with ordinary B-Si, the absorption spectrum is broadened from 400–1100 nm to 400–2500 nm, and the absorption is increased from 90.1 to 97.8% at 1100–2500 nm. It is possible to use the B-Si materials in the field of NIR-enhanced photoelectric detection and micro-optical night vision imaging due to the low cost, high compatibility, and reliability.

## Introduction

Silicon-based detectors have many advantages, such as a mature manufacturing process, low cost, and simple structure. Response bands of ordinary silicon-based detectors are mainly in the visible (VIS) band [1]. In order to enhance silicon-based materials absorption and realize the application of silicon-based materials in the NIR band, Mazur's team at Harvard University has successfully prepared new silicon-based materials with a surface micro–nano-trapped optical structure by using femtosecond laser irradiation of silicon-based material—black silicon [2, 3], which achieves high absorption. With the research development of B-Si materials, many new B-Si preparation methods are proposed, such as reactive ion etching, metal-assisted chemical etching, wet chemical etching, electrochemical etching, and other mainstream semiconductor processing methods [4–8]. Although these new methods have high prepared efficiency, the prepared B-Si materials are usually non-uniform and the absorption in the band spectrum above 1100 nm decreases rapidly. All of the above methods require sulfur-based element doping to enhance NIR absorption, but the instability of the complex doping process may result in the under-doping of sulfur-based elements [9, 10]. In recent years, many researchers devote to enhancing the optical properties of materials by the use of LSPR, which makes tunable broadband absorption become possible by preparing metallic metamaterials or random metal nanoparticles on the silicon surface [11, 12]. Some researchers have used Ag nanoparticles combined with micro–nano-structured silicon prepared by femtosecond laser to obtain materials with high absorption properties [13, 14], but there are problems that Ag is easily oxidized which leads to absorption attenuation of LSPR, and the resonance band of Ag nanoparticles is mainly concentrated in the VIS band [15]. In addition, AuNPs combined with micro–nano-structured silicon prepared by reactive ion etching enable a strong coupling of light to improve the optical properties of silicon-based materials, but there is still room for improvement [16, 17]. It inspires us to prepare micro–nano-structured B-Si by the use of femtosecond laser ablation and deposit AuNPs on its surface. The LSPR of metal nanoparticles excited by the light field [18] broadens the absorption spectrum of B-Si materials. Finally, the high absorption of VIS–NIR light based on silicon materials is realized, while overcoming the problem of NIR absorption attenuation caused by insufficient doping of sulfur-based elements.

In this paper, we proposed to combine the preparation of B-Si by femtosecond laser ablation with AuNPs to improve the NIR absorption of silicon. Compared with the preparation of B-Si by reactive ion etching, metal-assisted chemical etching, and other methods, the micro–nano-structure of B-Si prepared by femtosecond laser etching is more controllable, and the doping of sulfur elements can be brought in by femtosecond laser ablation during the condensation process after melting. Au has better oxidation resistance, and the resonance absorption peak is closer to the NIR band compared with Ag. We use the best preparation method of B-Si and the most suitable for infrared absorption of nanoparticles, to achieve high absorption of silicon-based materials in the VIS–NIR band. In addition, the absorption attenuation of B-Si materials in the NIR band has not been explained in detail in previous studies. We explain the reason for the decrease in absorptivity of B-Si materials in the NIR band and propose to use the LSPR effect of AuNPs to solve this problem. Finally, the absorption mechanism of B-Si composited with AuNPs is explained theoretically, and the models which conform to the actual structure of B-Si are simulated. The micro–nano-structured B-Si is prepared by irradiating the surface of silicon samples with a femtosecond pulsed laser under the SF_6_ atmosphere, and the AuNPs self-assembly on B-Si due to the self-shadow effect [19]. Broad-spectrum and high-absorption nanometer B-Si composited with AuNPs materials is obtained.

## Methods

Firstly, micro–nano-structured B-Si was prepared by femtosecond laser ablation, and the problem of NIR absorbance attenuation was found. The specific preparation method was as follows: In the first step, 6-inch intrinsic (111) silicon wafers with a resistivity of 3000–6000 Ω-cm were selected as substrates. The RCA standard cleaning method was used to clean silicon substrates, then, they were soaked in 5% hydrofluoric acid solution for 1 min and ultrasonically rinsed with deionized water for 10 min, and finally, silicon wafer surface contaminants were removed by 99.99% nitrogen. Cleaned silicon substrates were put into a vacuum cavity, the air inside the cavity was extracted, and then, SF_6_ gas was filled. The gas pressure inside the cavity was kept at 8.5 × 10^4^ Pa. In this paper, a titanium sapphire femtosecond laser was used to control the power density of 6.6 kJ/m^2^, which had a laser pulse center wavelength of 800 nm, a pulse width of 100 fs, and a pulse repetition frequency of 1 kHz. The scanning speed was 1 mm/s [10], and the scanning pitch was 0.01 mm. The prepared B-Si was placed in 5% hydrofluoric acid for 5 min and then ultrasonically cleaned with deionized water for 10 min, and the contaminants on the surface of B-Si were removed by 99.99% nitrogen. The morphology of micro–nano-structured B-Si was characterized by field emission scanning electron microscopy (SEM). The element content of materials was measured by energy-dispersive spectroscopy (EDS). The reflectance (R) and transmittance (T) of the samples were measured in the wavelength between 400 and 2500 nm by using a UV–VIS–NIR spectrophotometer (Shimadzu UV-3600i Plus). Before the test, a layer of BaSO_4_ was uniformly coated on the inner surface of the integrating sphere, so that the reflectance of the inner surface of the integrating sphere was close to 100% in the whole wide spectrum. The absorptance (A) was calculated via the formula A = 1– R − T.

**Fig. 1:**
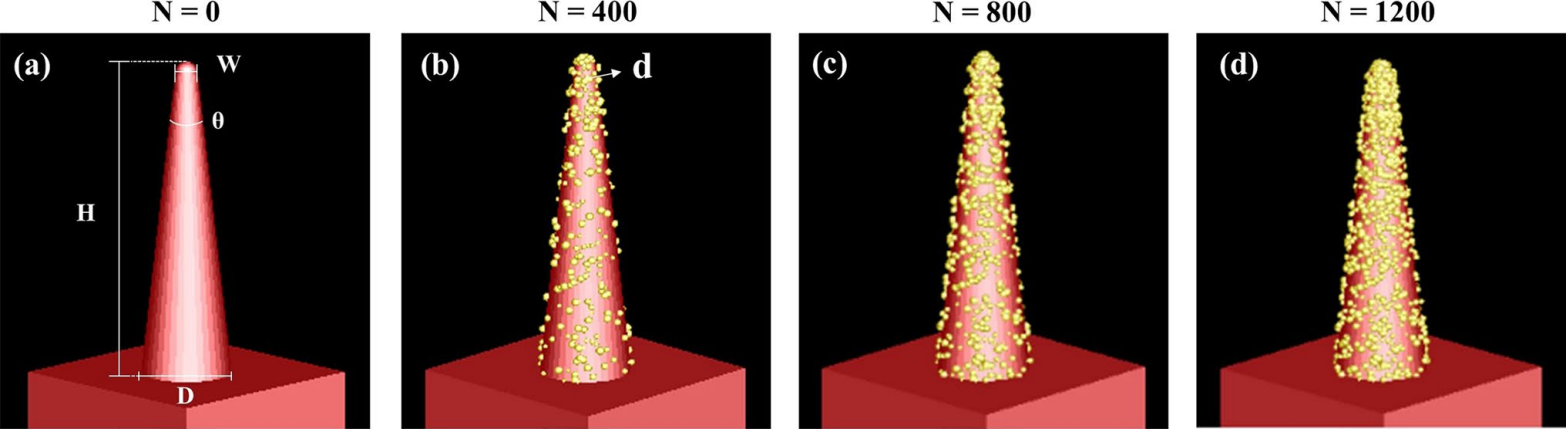
Models of B-Si composited with different numbers of AuNPs: **a** *N* = 0 (without AuNPs), **b** *N* = 400, **c** *N* = 800, **d** *N* = 1200

In order to solve the problem of NIR absorbance attenuation of silicon-based materials, we proposed a method of preparing AuNPs on the surface of B-Si. The optical absorption theory of metal nanoparticles and semiconductors was studied. Based on the absorption mechanism, the following simulation experiments were carried out: According to the actual pattern of B-Si formed by femtosecond laser ablation, the models of B-Si composited with AuNPs were established in finite-difference time domain (FDTD). As shown in Fig. 1, the B-Si model had a spike height *H* = 3 μm, spike angle *θ* = 10°, spike width *W* = 0.1 μm, base diameter *D* = 1 μm, AuNPs diameter *d* = 10– 25 nm, and the number of AuNPs *N* = 800–1200. The models with different diameters and numbers of AuNPs attached to the B-Si were simulated.

Finally, we experimented with depositing AuNPs on the micro–nano-structured B-Si. The preparation of 25-nm AuNPs onto B-Si was as follows: The B-Si prepared by femtosecond laser ablation was placed in thermal evaporation apparatus with a vacuum evaporation chamber air pressure of 10^–3^ Pa, the B-Si substrate temperature of 350 K, a sputtering power of 800–6000 W, and an average deposition rate of 0.5 nm/s. The performance of B-Si composited with AuNPs materials was tested by the same test method as that of ordinary B-Si. The experimental results were in agreement with the theoretical analysis and simulation results, and the problem of NIR absorbance attenuation was solved.

## Results and discussion

Compared with the preparation of B-Si by reactive ion etching, metal-assisted chemical etching, and other methods, the micro–nano-structure of B-Si prepared by femtosecond laser etching is more controllable, and the doping of sulfur elements can be brought in by femtosecond laser ablation during the condensation process after melting. The energy band of impurity in B-Si semiconductors makes the forbidden bandwidth of silicon narrower and facilitates the absorption of photons with energy less than the forbidden bandwidth. It also breaks the limitation of the silicon-based materials in the infrared absorption and broadens the NIR absorption spectral range. Using a femtosecond laser to ablate silicon in an SF_6_ atmosphere to achieve sulfur doping which can form an impurity energy band and B-Si micro–nano-structure, the reaction process [20] is:

1 $${\text{SF}}_{6} \left( {\text{g}} \right) \, + {\text{ Si}}\left( {\text{s}} \right) \to {\text{SF}}_{6} - {\text{Si}}\left( {{\text{ads}}} \right)$$

2 $${\text{SF}}_{6} - {\text{Si}}\left( {{\text{ads}}} \right) \, \to {\text{ SiF}}_{2} \left( {{\text{ads}}} \right) \, + {\text{ SF}}_{4} \left( {\text{g}} \right) \uparrow$$

3 $${\text{SF}}_{6} \left( {\text{g}} \right) + {\text{SiF}}_{2} \left( {{\text{ads}}} \right) \to {\text{SiF}}_{4} \left( {{\text{ads}}} \right) + {\text{SF}}_{4} \left( {\text{g}} \right) \uparrow$$

4 $${\text{SiF}}_{4} \left( {{\text{ads}}} \right) \to {\text{SiF}}_{4} \left( {\text{g}} \right) \uparrow$$

Femtosecond laser pulses irradiate SF_6_ gas on the silicon wafer surface, causing ionization and decomposition of SF_6_ gas and meanwhile ultrafast melting and condensation on the surface of the silicon wafer. Sulfur atoms generated by the decomposition of SF_6_ gas are bound with the silicon surface to form supersaturated doping. Multiple impurity energy levels are generated in the band gap of silicon superimpose on each other to form impurity energy bands [21, 22] and intersect with the silicon conduction band bottom overlap, thus reducing the forbidden bandwidth of silicon. Incident photons with photon energy less than the forbidden bandwidth can also be absorbed, as shown in Fig. 8, stage 1*:

5 $$E_{i} - E_{V} < h\nu \, < E_{g} = E_{C} - E_{V}$$

From (5) to (8), it can be seen that the limited absorption wavelength *λ*_0_ is extended to the NIR band, enhancing the absorption capacity of the materials in the NIR band:

6 $$h\nu_{0} = E_{i} - E_{V} < E_{g}$$

7 $$\nu_{0} = \frac{c}{{\lambda_{0} }}$$

8 $$\lambda_{0} (\mu m) = \frac{1.24}{{E_{i} - E_{V} }} > \frac{1.24}{{E_{g} }}$$

where *h* = 6.626 × 10^–34^ J·s, *c* = 3 × 10^8^ m/s, and *E*_*g*_ = 1.12 eV.

We previously investigated the effects of laser power, gas pressure, and scanning speed on the morphology and absorption rate of B-Si [10]. It was found that experimental conditions often cause the problem of insufficient doping of sulfur-based elements. In this work, experiments with samples located at different distances from the laser focus and multiple cycles of scanning are conducted, and the results are given in the following.

**Fig. 2:**
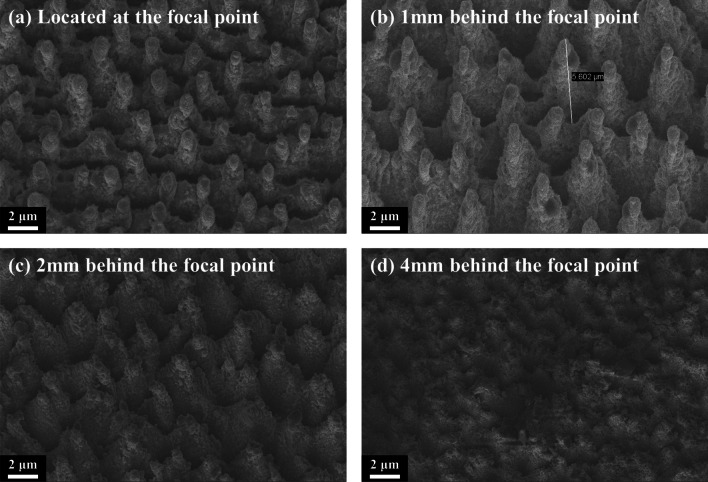
Surface morphology SEM of B-Si formed by femtosecond laser ablation at different distances between the sample and focus of the laser beam: **a** located at the focal point; **b** 1 mm behind the focal point; **c** 2 mm behind the focal point; **d** 4 mm behind the focal point

The micro–nano-structure of B-Si determines its optical absorption performance. B-Si is formed by femtosecond laser ablation under conditions: gas pressure 8.5 × 10^4^ Pa, power density 6.6 kJ/m^2^, scanning speed 1 mm/s, and scanning pitch 0.01 mm. As shown in Fig. 2, the femtosecond laser ablates in one cycle, varying the distance between the sample and focus of the laser beam to control the ablation micro–nano-structured B-Si morphology. As shown in Fig. 2a, when the sample surface is located at the focus of the laser beam, the micro–nano-structure is cylindrical. This is because the high energy density of the laser at the focal point leads to excessive melting of the sample. As shown in Fig. 2b, the laser energy density that radiates to the surface of the sample is uniform when it is 1 mm behind the focal point; thus, a regular spiky cone-shaped structure is formed. As shown in Fig. 2c, d, as the sample surface moves away from the focus of the laser beam, the laser energy intensity becomes weaker, and the state of insufficient melting appears. The spiky cone-shaped structure cannot be fully shaped, and it is replaced by a rough and messy shape of the surface.

**Fig. 3:**
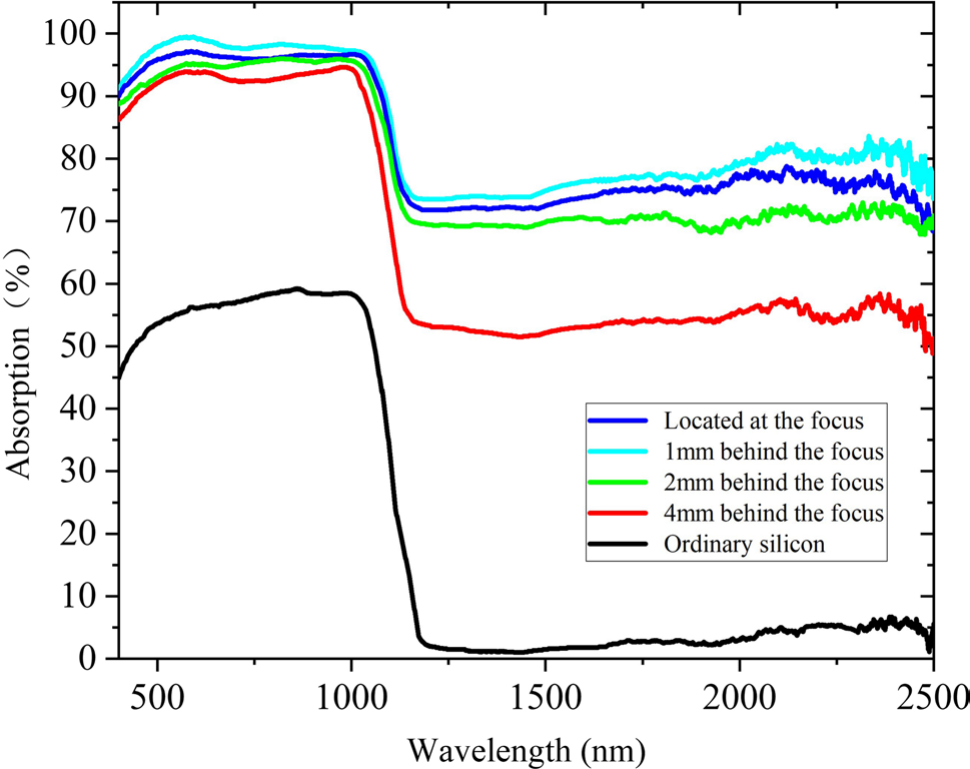
Absorption curves of B-Si formed by femtosecond laser ablation at different distances between the sample and focus of the laser beam

As shown in Fig. 3, absorption curves of micro–nano-structured B-Si formed by femtosecond laser ablation at different distances between the sample and focus of the laser beam are tested. It can be seen that the absorption of ordinary silicon is not more than 60% at 400–1100 nm and almost no absorption at wavelengths greater than 1100 nm. However, the absorption of B-Si obtained by femtosecond laser ablation is improved. The micro–nano-structured B-Si has the highest average absorption at the position 1 mm behind the focal point, and the average absorption in the range of 400–1100 nm and 1100–2500 nm is 97.2% and 77.4%, respectively.

**Fig. 4:**
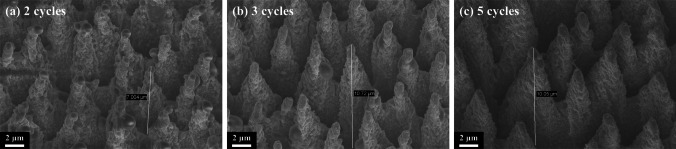
Surface morphology SEM of B-Si formed by femtosecond laser ablation at different cycles: **a** two cycles, **b** three cycles, **c** five cycles

As shown in Fig. 4, micro–nano-structured B-Si morphology at 1 mm behind the focal point under different cycles is presented. It can be seen that the height of the B-Si spike is increased to about 10 μm under five cycles, the surface becomes smoother, and the spike cone-shaped became more pronounced as the cycle increases.

**Fig. 5:**
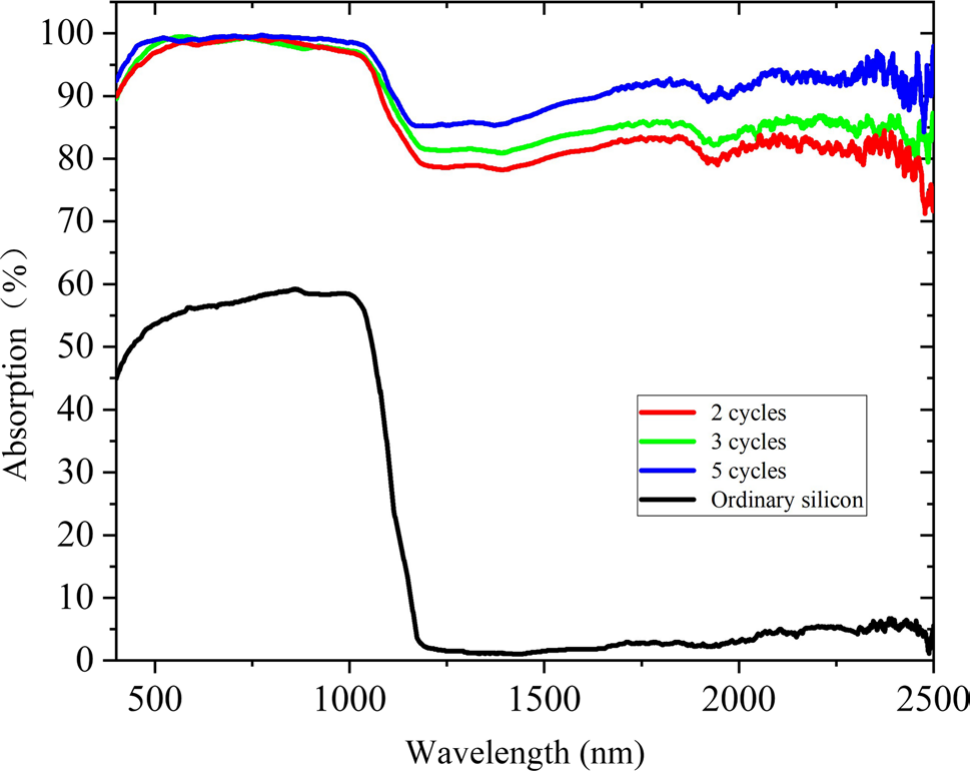
Absorption curves of B-Si formed by femtosecond laser ablation at different cycles

As shown in Fig. 5, absorption curves of micro–nano-structured B-Si formed by different cycles of femtosecond laser ablation are tested. The absorption of the B-Si increases as the cycle increases, and the highest average absorption is obtained after five cycles; the average absorption in the range of 400–1100 nm and 1100–2500 nm is 98.5% and 90.1%, respectively. However, the micro–nano-structured B-Si prepared by the traditional method usually shows NIR absorbance attenuation.

**Fig. 6:**
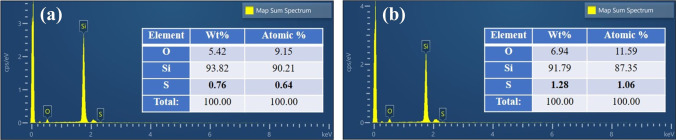
EDS of B-Si formed by femtosecond laser ablation at different cycles: **a** one cycle, **b** five cycles

As shown in Fig. 6, the surface EDS of micro–nano-structured B-Si formed by one cycle and five cycles of ablation is tested. The elemental sulfur content of the B-Si increases after five cycles of ablation, and the corresponding absorption is higher. The mass ratio and atomic ratio of the elemental sulfur on the B-Si surface after five cycles of ablation are 1.28% and 1.06%, respectively. Despite the multiple cycles of femtosecond laser scanning ablation, the sulfur doping concentration of B-Si remains low. It still faces the problem of NIR absorption attenuation.

From the above experimental results, the doping of sulfur-based elements in micro–nano-structured B-Si achieves the absorption of NIR light. There are two reasons for the absorption increases as the cycle increases, and the highest average absorption is obtained after five cycles. Firstly, the peak height of B-Si prepared by five cycles of ablations reaches 10 μm. This higher and denser trapping micro–nano-structure increases the reflection times of incident light between peak structures, thus reducing the surface reflectance of B-Si. Second, with the increase in cyclic, the content of doped sulfur in B-Si increases. This results in more impurity levels in the energy band of B-Si, which increases the absorption of light in the NIR band. It usually brings the problem of insufficient doping of sulfur-based elements due to the limited doping process, which leads to the NIR absorption attenuation of B-Si materials. To solve this problem, a method of preparing AuNPs on the surface of B-Si is proposed. The strong resonant absorption of AuNPs of arbitrary size and shape in the VIS–NIR band is used to broaden the absorption spectrum and enhance the absorption of silicon-based materials. Next, the absorption mechanism of the micro–nano-structured B-Si composited with AuNPs is investigated.

**Fig. 7:**
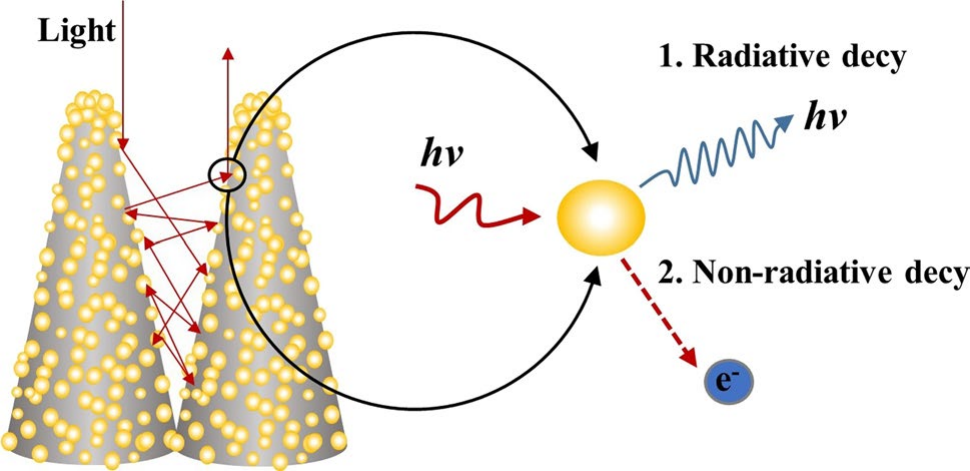
Theoretical model of B-Si composited with AuNPs

Since Au has better oxidation resistance, the resonance absorption peak is closer to the NIR band compared with Ag. We use the best preparation method of B-Si and the most suitable for infrared absorption of nanoparticles, to achieve high absorption of silicon-based materials in the VIS–NIR band. As shown in Fig. 7, the spiky cone-shaped B-Si structure with AuNPs attached makes the surface of the B-Si micro–nano-structure more complex and increases the reflection of light in the micro–nano-structure, which is conducive to the absorption of incident light. At the same time, the incident light energy decays in two forms in femtosecond time. One is the radiation decay process of scattering consumption by re-emitting photons outward, and the re-emitted photons can be absorbed by the complex micro–nano-structure for secondary collection. The other one is that the non-radiative decay process transfers energy to free electrons to form hot electrons. When free electrons in the metal can absorb photon energy, that is, *hν* > *qф*_*N-P*_ (*ф*_*N-P*_ is the electron escape work of metal nanoparticles), they will ionize to form photoelectrons to escape from the metal surface. When *hν* < *qф*_*N-P*_, the excited electrons leap to high energy levels and are bound in the metal, and these electrons in excited states are called hot electrons [23, 24]. The photon absorption efficiency of the metal is the main factor to determine the efficiency of hot electron generation.

When light is irradiated on the arbitrary shape and size of metal nanoparticles, the free electrons inside it interact with the incident light and appear to co-oscillate. At this time, the photons matching this resonant frequency will be scattered or absorbed. The local electric field appears to be significantly enhanced, called the LSPR of nanoparticles [18]. Since the resonance wavelength of AuNPs can cover the VIS–NIR bands, hot electrons may be collected and absorbed by B-Si material through the barrier between the metal and B-Si material.

**Fig. 8:**
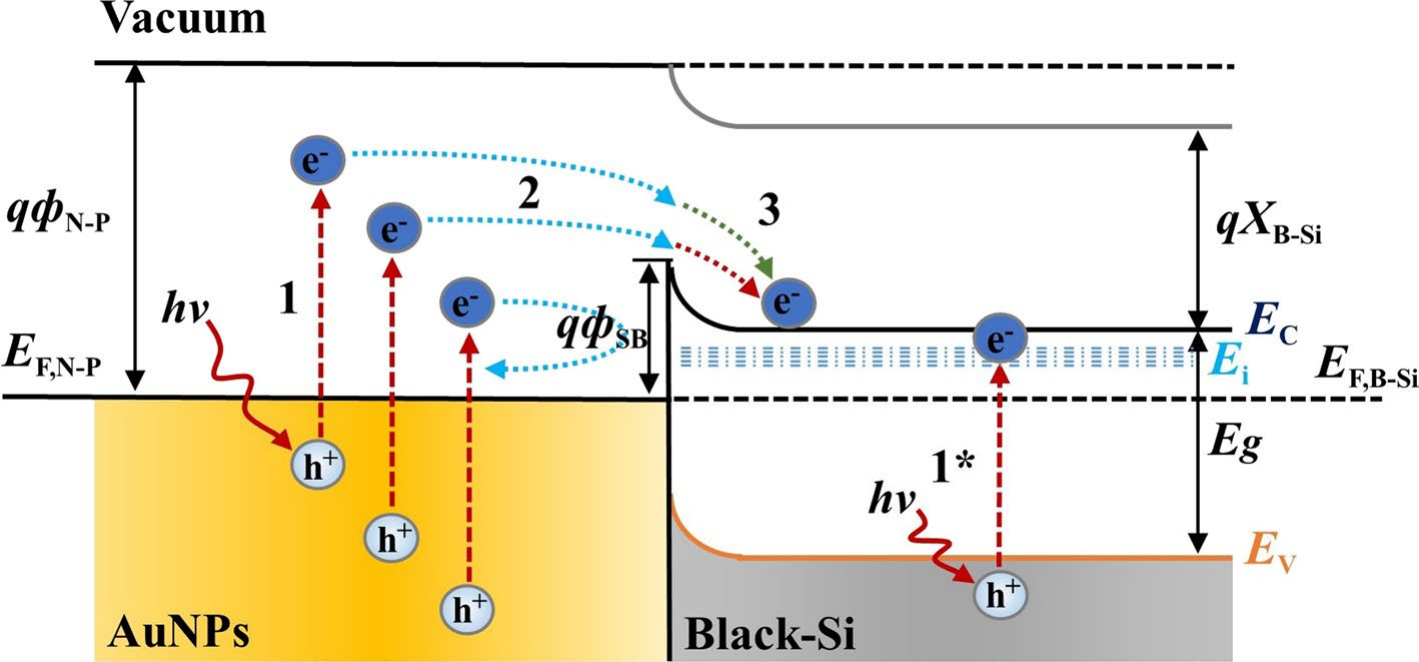
Light absorption theory of B-Si composited with AuNPs (*E*_*F, N-P*_: Fermi energy level of metal nanoparticles; *E*_*F, B-Si*_: Fermi energy level of B-Si; *ф*_*N-P*_: electron escape work of metal nanoparticles; *ф*_*SB*_ = *ф*_*N-P*_—*X*_*B-Si*_: Schottky barrier; *X*_*B-Si*_: electron affinity potential of B-Si semiconductor; *E*_*C*_: conduction band; *E*_*V*_: valence band; *E*_*i*_: impurity energy band; *E*_*g*_ = *E*_*C*_*—E*_*V*_*:* forbidden bandwidth)

As shown in Fig. 8, the collection process of hot electrons generated by LSPR of metal nanoparticles is divided into three main stages: 1. Hot electrons are excited; 2. hot electrons transfer to the barrier interface; and 3. hot electrons cross the barrier into the semiconductor. In the first stage, the nano-metal particles produce LSPR under illuminated conditions, and the incident light transfers energy to the free electrons to form hot electrons. When the electrons are excited to form hot electrons, they will enter stage 2. The hot electrons are generated in various parts of the nano-metallic structure, finally moving into the metal and reaching the Schottky barrier interface. However, since the initial direction of hot electrons is uncertain, not all hot electrons can reach the barrier surface. In theory, hot electrons with higher energy are more likely to reach the barrier interface. The hot electrons that reach the barrier interface will enter stage 3, because of the low probability that the hot electrons tunnel directly at the interface, the hot electrons require enough kinetic energy to overcome the Schottky barrier *ф*_*SB*_ to enter the semiconductor smoothly for collection. If the hot electrons do not have enough energy to tunnel the *ф*_*SB*_, they will eventually be converted to Joule heat within the metal. As in the above process, hot electrons must undergo complex screening before they finally reach the B-Si materials to be collected successfully [25, 26].

Next, simulation models which conform to the actual structure of B-Si are established according to the absorption mechanism of the B-Si composited with AuNPs. The absorption of models with different diameters and numbers of AuNPs attached to the B-Si is simulated, and the results are given in the following.

**Fig. 9:**
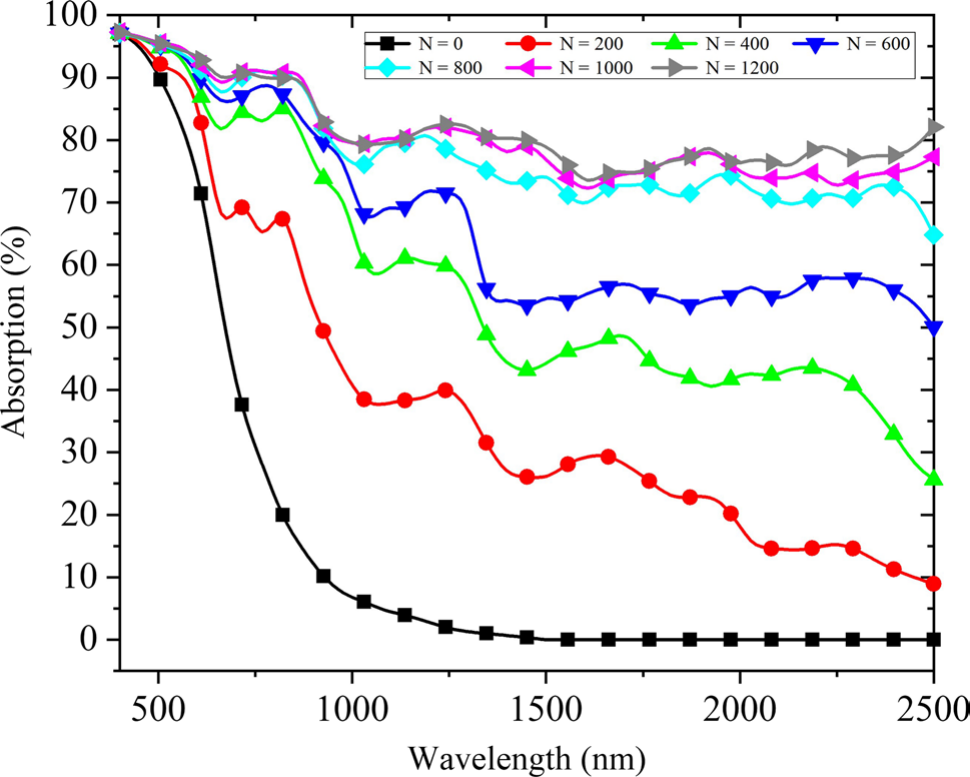
Absorption curves of B-Si composited with 10–25-nm AuNPs randomly distributed (particle numbers of 0, 200, 400, 600, 800, 1000, 1200)

As shown in Fig. 9, absorption curves of micro–nano-structured B-Si composited with 10–25-nm AuNPs randomly distributed are simulated. For B-Si without sulfur-based element doping, the absorption attenuation is obvious in the NIR band and almost no absorption at wavelengths greater than 1100 nm. However, the B-Si surface covered with AuNPs shows an absorption. When AuNPs diameter is fixed at *d* = 10 nm ~ 25 nm, the absorption efficiency of spiky cone-shaped B-Si gradually increases as the number of covered particles increases. When the number of AuNPs is increased to N = 800, the average absorption in 400–1100 nm is 87.7%, and the average absorption in 1000–2500 nm is 72.6%. Due to the limitation of the surface area of B-Si models, increasing the number of AuNPs will cause adjacent nanoparticles to adhere and form a metal film, leading to a slower increase in absorption. When the number of AuNPs is increased to *N* = 1200, the average absorption in 400–1100 nm is 88.5%, and the average absorption in 1000–2500 nm is 78.0%. Covering AuNPs on the surface of B-Si can enhance the absorption of B-Si in the NIR band, and the absorption increases with the increase of AuNPs quantity, but the increase of absorption gradually becomes slower after reaching a certain AuNPs quantity. The micro–nano-structure of B-Si covered with AuNPs has higher absorption and a wider absorption spectrum.

**Fig. 10:**
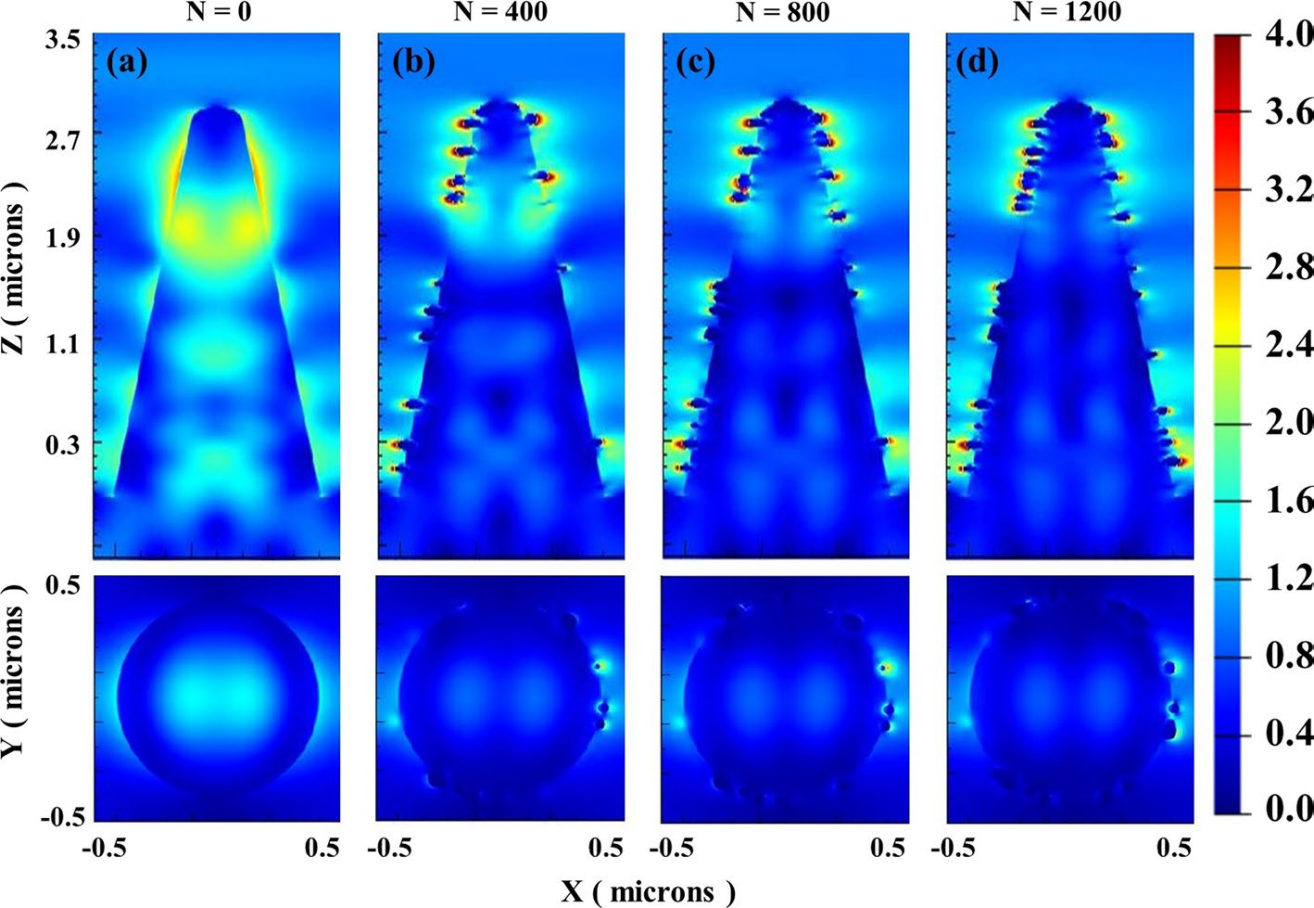
Electric field distribution of (*X*–*Z*) cross section and (*X*–*Y*) cross section of B-Si composited with 10–25-nm AuNPs randomly distributed under the incident wavelength of 1550 nm: **a** *N* = 0 (without AuNPs), **b** *N* = 400, **c** *N* = 800, **d** *N* = 1200

As shown in Fig. 10, the electric field distribution of the (*X*–*Z*) cross section and (*X*–*Y*) cross section of micro–nano-structured B-Si composited with 10–25-nm AuNPs randomly distributed under the incident wavelength of 1550 nm is simulated. 10–25-nm AuNPs that are randomly distributed on the B-Si surface form various styles of AuNPs aggregates, causing hybrid LSPR patterns and eventually expanding the resonance absorption spectrum. The electric field energy of B-Si composited with AuNPs at 1550 nm is concentrated on the B-Si spike surface, and more hot spots appear on the B-Si surface as the number of AuNPs increases. The micro–nano-structured B-Si composited with AuNPs had stronger NIR absorption, and this enhanced absorption also occurs at arbitrary wavelengths from 1100 to 2500 nm.

**Fig. 11:**
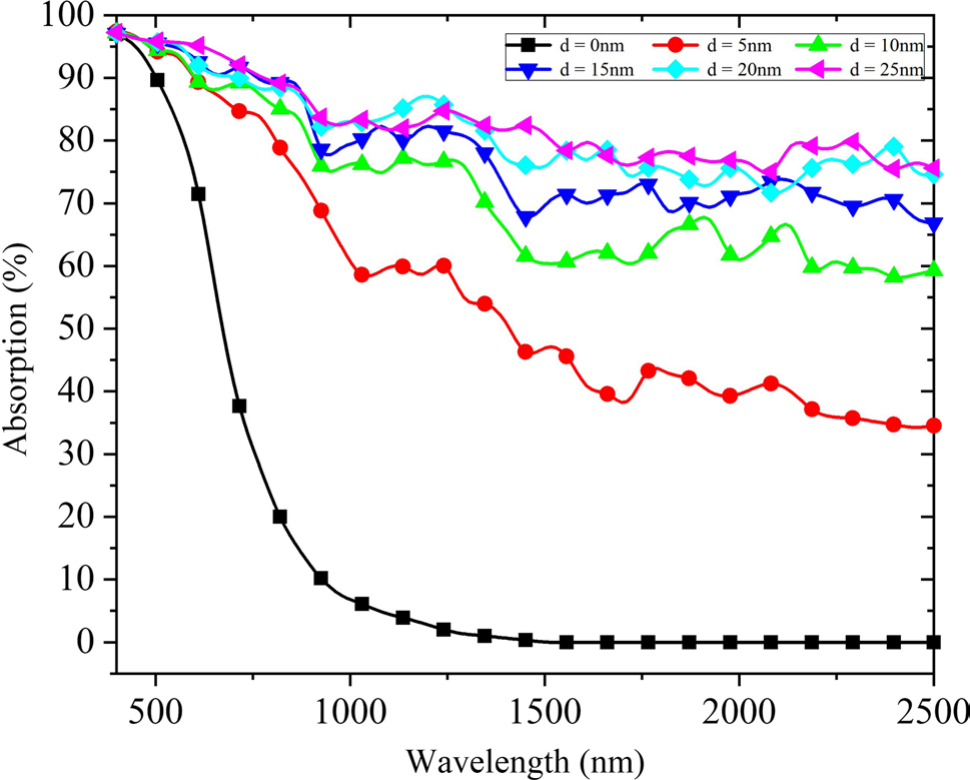
Absorption curves of B-Si composited with 1000 AuNPs randomly distributed (particle diameter of 0 nm, 5 nm, 10 nm, 15 nm, 20 nm, 25 nm)

As shown in Fig. 11, absorption curves of micro–nano-structured B-Si composited with 1000 AuNPs randomly distributed are simulated. It can be observed that the absorption of B-Si composited with AuNPs gradually increases as the diameter of AuNPs under fixed AuNPs number *N* = 1000 increases. When the AuNPs diameter *d* = 25 nm, the average absorption in 400–1100 nm is 89.8%, and the average absorption in 1000–2500 nm is 78.9%. The NIR absorption attenuation of undoped sulfur-based element B-Si is improved effectively.

From the above simulation results, for micro–nano-structured B-Si without sulfur-based element doping, the absorption decays significantly in the NIR band and almost no absorption at wavelengths greater than 1100 nm. By adding AuNPs with different diameters randomly distributed on their surface, the absorption spectrum of silicon-based materials can be broadened and the absorption in VIS–NIR bands can be enhanced at the same time. It effectively improves the NIR absorbance attenuation caused by insufficient doping of sulfur-based elements. Moreover, the absorption gradually increases as the number and diameter of AuNPs increase, and the absorption is saturated after reaching a certain number and diameter of AuNPs. Next, experiments of micro–nano-structured B-Si composited with AuNPs are conducted.

**Fig. 12:**
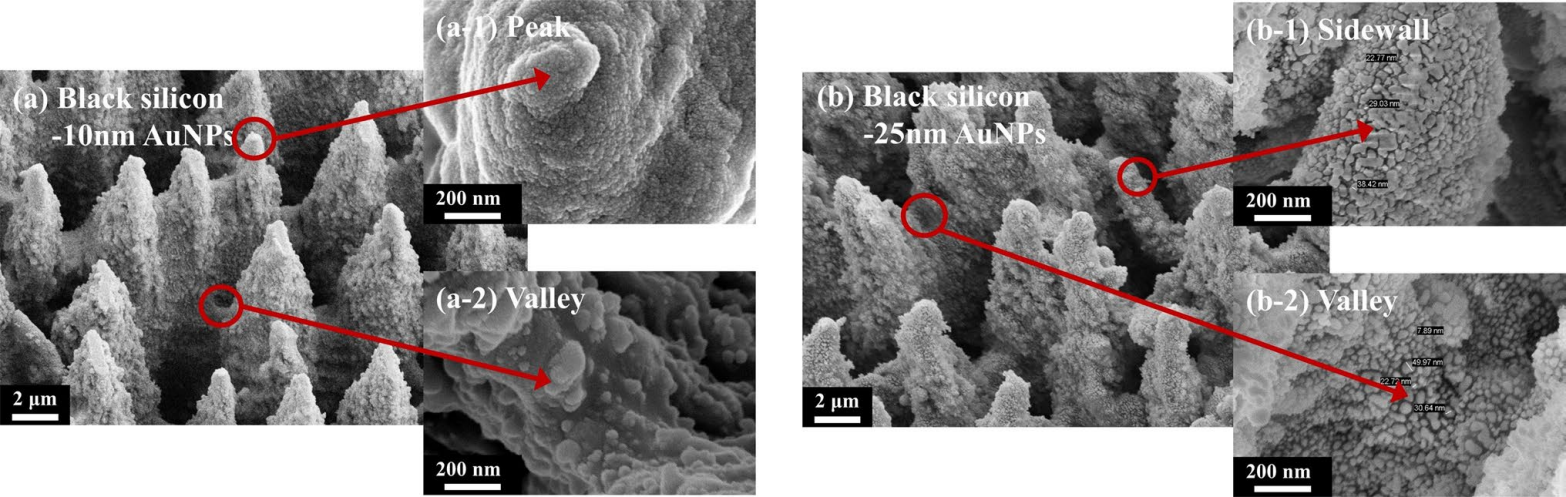
Surface morphology SEM of B-Si deposited with different thicknesses of AuNPs (B-Si formed by five-cycle femtosecond laser ablation): **a** B-Si composited with 10-nm AuNPs; **a-1** peak of B-Si composited with 10-nm AuNPs; **a-2** valley of B-Si composited with 10-nm AuNPs; **b** B-Si composited with 25-nm AuNPs; **b-1** sidewall of B-Si composited with 25-nm AuNPs; **b-2** valley of B-Si composited with 25-nm AuNPs

As shown in Fig. 12, the surface morphology of micro–nano-structured B-Si deposited with 10-nm AuNPs and 25-nm AuNPs is observed by SEM. As shown in Fig. 12a, Au particles on the surface of B-Si deposited with 10-nm AuNPs are uniform and unaggregated. As shown in Fig. 12b, Au particles on the surface of B-Si deposited with 25-nm AuNPs show aggregation at the top and bottom. There are randomly distributed and arbitrarily shaped Au particles in the size range of 10–50 nm on the sidewall.

**Fig. 13:**
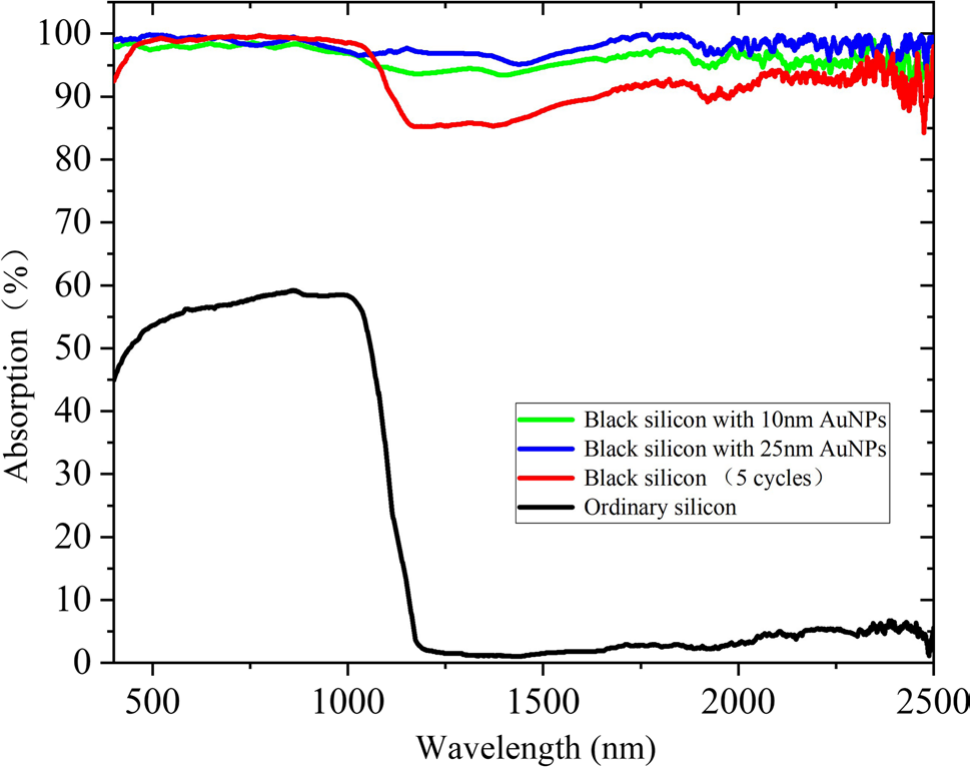
Absorption curves of B-Si deposited with 10-nm and 25-nm AuNPs (micro–nano-structured B-Si formed by five-cycle femtosecond laser ablation)

As shown in Fig. 13, absorption curves of nanometer B-Si deposited with 10-nm AuNPs and 25-nm AuNPs are tested. The absorption of AuNPs-covered B-Si is significantly enhanced in 1100–2500 nm. The absorption of B-Si deposited with 25-nm AuNPs is higher, with an average absorption of 98.6% from 400 to 1100 nm and 97.8% from 1100 to 2500 nm. Compared with B-Si without AuNPs, the absorption is increased from 90.1 to 97.8% at 1100–2500 nm, which effectively improves the NIR absorbance attenuation. If the size of AuNPs keeps increasing, the absorption of materials can be improved to some extent. But it can be seen from the simulation and experimental results that there is little room for improving the absorption. When the size of AuNPs increases to a certain extent, continuous Au films will be formed on the surface by further deposition of AuNPs. It will reflect the incident light and weaken the optical absorption of materials. The B-Si composited with 25-nm AuNPs is an ideal parameter obtained by simulation and experiment.

## Conclusions

In this paper, we successfully prepare micro–nano-structured B-Si by the use of femtosecond laser ablation and deposit AuNPs on its surface. LSPR of AuNPs excited by light field is used to achieve B-Si materials with broad spectrum and high absorption. Experimental results show that the average absorption of nanometer B-Si composited with 25-nm AuNPs in the range of 400–1100 nm and 1100–2500 nm is 98.6% and 97.8%, respectively. Compared with ordinary B-Si, the absorption spectrum is broadened from 400–1100 nm to 400–2500 nm, and the absorption is increased from 90.1% to 97.8% at 1100–2500 nm. It effectively improves the NIR absorbance attenuation caused by insufficient doping of sulfur-based elements. It is promising to use the B-Si materials in the field of NIR-enhanced photoelectric detection and micro-optical night vision imaging.

## Data Availability

The datasets used or analyzed during the current study are available from the corresponding author upon reasonable request.
